# CD34^dim^ cells identified as pluripotent stem cell‐derived definitive hemogenic endothelium purified using bone morphogenetic protein 4

**DOI:** 10.1111/cpr.13366

**Published:** 2022-12-07

**Authors:** Soo‐Been Jeon, A‐Reum Han, Sunghun Lee, Seung Chan Lee, Min Ji Lee, Soon‐Jung Park, Sung‐Hwan Moon, Ji Yoon Lee

**Affiliations:** ^1^ CHA Advanced Research Institute, Bundang CHA Medical Center CHA University Seongnam Kyunggi‐do Republic of Korea; ^2^ R&D Division CHA Biotech Seongnam Kyunggi‐do Republic of Korea; ^3^ Research Institute T&R Biofab Co. Ltd Siheung Republic of Korea; ^4^ Department of Medicine Konkuk University School of Medicine Seoul Republic of Korea; ^5^ Department of Animal Science and Technology Chung‐Ang University Anseong‐si Republic of Korea; ^6^ Department of Biomedical Science CHA University Seongnam Kyunggi‐do Republic of Korea

## Abstract

Hemogenic endothelium (HE) plays a pivotal and inevitable role in haematopoiesis and can generate all blood and endothelial lineage cells in the aorta‐gonad‐mesonephros of mouse embryos. Whether definitive HE can prospectively isolate pure HE from human pluripotent stem cells that can spontaneously differentiate into heterogeneous cells remains unknown. Here, we identified and validated a CD34^dim^ subpopulation with hemogenic potential. We also purified CD34 cells with a CXCR4^−^CD73^−^ phenotype as a definitive HE population that generated haematopoietic stem cells and lymphocytes. The frequency of CXCR4^−^CD73^−^CD34^dim^ was evidently increased by bone morphogenetic protein 4, and purified HE cells differentiated into haematopoietic cells with myeloid and T lymphoid lineages including Vδ2+ subset of γ/δ T cells. We developed a simple method to purify HE cells that were enriched in CD34^dim^ cells. We uncovered an initial step in differentiating haematopoietic lineage cells that could be applied to basic and translational investigations into regenerative medicine.

## Graphical abstract

Purified hemogenic endothelium (HE) was successfully isolated in pluripotent stem cells at Day 5. Definitive HE which is defined by CXCR4^−^CD73^−^ CD34^+^ cells, was enriched in CD34^dim^ population. CXCR4^−^CD73^−^ phenotype was enriched in most CD34 cells including CD34^dim^ and CD34^bright^ population on Day 5, which is optimal day to isolate CD34 HE.

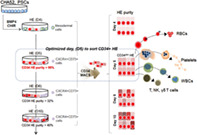

## 1 INTRODUCTION

Haematopoietic cells, including myeloid and lymphoid lineage cells in early embryos, originate from definitive hemogenic endothelium (HE) via a process called the endothelial‐to‐haematopoietic transition (EHT).^1^, ^2^ The embryonic haematopoietic system includes distinct programming for cell potential and developmental properties. One is primitive haematopoiesis, which initially emerges in the yolk sac and has a restricted capacity to differentiate into primitive erythroblasts, monocytes and megakaryocytes.^3^ The other is definitive haematopoiesis, which follows a primitive haematopoiesis stage that is autonomously initiated by the embryonic aorta‐gonad‐mesonephros (AGM) region and has functional haematopoiesis including haematopoietic stem cells (HSCs) and lymphoid lineage cells.^4^ These findings have been mostly validated in studies of mouse embryogenesis and development. Similar with mouse embryo development, the differentiation of functional blood lineage progenitors from pluripotent stem cells (PSCs) is also initiated by HE specification. Although establishing functional HE in vitro is pivotal to generating haematopoietic lineage cells, studies of the haematopoietic specification are hampered by a lack of consensus on the timing of HE analyses and the full haematopoietic potential of this population. Human HE cells derived from PSCs comprise a transient endothelium with immunophenotypes such as the cluster of differentiation (CD)31, cadherin (CDH)5, kinase insert domain‐containing receptor (KDR), and tunica interna endothelial cell kinase (TIE2) without other lineage markers such as CXCR4, CD73, CD45, and the 235a phenotype.^5^, ^6^, ^7^ Such HE mainly comprises CD34^+^ cells with the CXCR4^−^CD73^−^ phenotype and KDR gives rise to endothelial lineage cells and haematopoiesis due to having bipotentiality.^8^, ^9^ The concept of HE as a reservoir of all haematopoietic cells in the AGM region has been investigated; however, little is known about lymphoid lineage cells and HSCs, which are restricted in definitive but not in primitive HE, during development.^4^, ^9^

Bone morphogenetic protein (BMP) 4 stimulates the differentiation of mesoderm lineages, including the haematopoietic lineage and HE from PSCs.^10^, ^11^, ^12^ Furthermore, BMP4 plays an indispensable role in the proliferation of haematopoietic lineage progenitors.^13^ Stepwise dissection of whole embryos at the molecular level has recently shown that BMP signalling in *Xenopus* is closely associated with hemangioblast formation, but not the specification of HE, which is different from that between mice in humans.^14^ This led to a debate about whether BMP4 is required to stabilize HE formation in differentiating human PSCs. Based on these findings, we examined the effects of various concentrations of BMP4 in definitive HE and investigated its role in HE development and haematopoietic lineage cells ex vivo. Understanding HE cells in the context of human development requires PSC‐derived HE, which finally undergoes EHT to generate haematopoietic progenitor cells, including T lymphocyte progenitors. Previous studies have mostly been retrospective; therefore, a prospective study of purified HE is required to further elucidate the progressive specification of lineage progenitors.^7^, ^15^ To date, most studies for HE is focused on identification with specific markers or role of pivotal transcription factors to commit HE.^3^, ^7^, ^9^, ^16^, ^17^, ^18^, ^19^ Most of these differentiation are successful in identifying the HE with detailed method, but despite much effort, protocol for prospective isolation of HE from PSCs have not yet been achieved due to PSC heterogeneity. The critical regulator SRY‐Box Transcription Factor 17 (Sox17) identifies bona fide HE cells that produce T lymphocytes and express the markers KDR, vascular endothelial (VE)‐cadherin, and CD31 that ultimately leads to haematopoiesis.^3^ Like the Sox family, including Sox17, several transcription factors such as Sox7, ETS Variant Transcription Factor 2 (ETV2), Proto‐Oncogene, AP‐1 transcription factor subunit (Fosb), and growth factor independence‐1 (Gfi1) have emerged as key factors in haematopoiesis.^18^, ^19^, ^20^ However, these transcription factors cannot function in the prospective isolation of HE cells as surface markers. To prospectively isolate HE, we investigated the ratio of CD34^dim^CXCR4^−^CD73^−^ HE cells daily under optimal conditions and ultimately determined the optimal time point and culture conditions to enrich CD34^dim^CXCR4^−^CD73^−^ HE cells using BMP4. Purified HE cells committed to haematopoietic lineage cells expressing pivotal transcriptional factors. These approaches that rely on mimicked aspects of ontogenetic processes collectively facilitated the isolation of pure HE from PSCs. Furthermore, purified HE cells might provide a platform for definitive academic studies of developmental biology and the discovery of clinically relevant clues in regenerative medicine.

## 2 MATERIALS AND METHODS

### 2.1 Human PSCs and magnetic‐activated cell sorting

All experiments proceeded under authorization from the Institutional Review Board for Human Research at the CHA University (1044308‐202204‐LR‐023‐02) for CHA52 and Konkuk University (KUH1280081 for H1 and H9). Undifferentiated human PSCs (CHA52, H1, H9 embryonic stem cell line, PSCs) were seeded onto Matrigel®‐coated plates with mTeSR™1 medium (85850; StemCell Technologies Inc.) to differentiate HE from PSCs. The cells were incubated with APEL™ 2 medium supplemented with 3 nM − 1.5 μM CHIR‐99021 (S2924, Selleck Chemicals), 20 ng/ml of vascular endothelial growth factor (VEGF)_165_ (100‐20; PeproTech Inc.), and 25 ng/ml HumanKine® BMP‐4 (HZ‐1045; Proteintech Group Inc.) for 3 days to induce mesoderm.^21^ Based on previous, to induce and expand the HE, cells were then incubated with APEL™ 2 medium supplemented with 25 ng/ml HumanKine® BMP‐4 (HZ‐1045, Proteintech), and 250 ng/ml stem cell factor (SCF; 300‐07), 20 ng/ml VEGF_165_ (100‐20), 200 ng/ml FMS‐like tyrosine kinase 3 (Flt3)‐Ligand (300‐19), 100 ng/ml Thrombopoietin (TPO) (300‐18) and 20 ng/ml erythropoietin (EPO) (100‐64; all from PeproTech) as described previous papers.^12^, ^17^, ^18^, ^22^, ^23^, ^24^, ^25^, ^26^ We isolated CD34^+^ cells from PSC‐derived HE by magnetic‐activated cell sorting (MACS) using CD34 MicroBead Kits (Miltenyi Biotec) as described by the manufacturer.^27^

### 2.2 Flow cytometry

Hemogenic endothelial cells (ECs) derived from PSCs were incubated with antibodies, washed with 0.5% FBS in PBS, and analysed using a BD Accuri C6 plus flow cytometer (BD Biosciences). The antibodies used in this study were as follows: FITC‐conjugated mouse anti‐human CD34 (348053, BD Biosciences), APC‐conjugated mouse anti‐human CDH5 (17‐1449‐41, eBioscience), goat anti‐human TIE2 (AF313, R&D Systems Inc.); PE‐conjugated mouse anti‐human CXCR4 (555974), FITC‐conjugated Mouse Anti‐Human CD45 (555482), APC‐conjugated mouse anti‐human CD90 (561971) and PE‐Cy™7‐conjugated mouse anti‐human CD235a (563666) (all from BD Pharmingen Inc.); APC‐conjugated mouse anti‐human CD73 (344006), PerCP/Cyanine 5.5‐conjugated mouse anti‐human CD41 (303720), APC‐conjugated mouse anti‐human CD43 (343206), PE‐conjugated mouse anti‐human CD3 (300408), PE‐conjugated mouse anti‐human TCR Vγ9 (331308) and FITC‐conjugated mouse anti‐human TCR Vδ2 (331406; all from BioLegend), Simultest™ FITC/PE‐conjugated anti‐human CD3/CD16^+^CD56^+^ (340042; BD Biosciences), and CD11b Monoclonal antibody (14‐0118‐82, eBioscience).

### 2.3 Immunocytochemistry

Human PSCs, HE, and differentiated cells were fixed with 2% PFA and permeabilized with 0.25% Triton X‐100 for 20 min at room temperature. The cells were then washed with PBS, non‐specific antibody binding was blocked with 5% normal goat serum, and the cells were incubated with primary, followed by secondary antibodies. Nuclei were stained with 4′, 6‐diamidino‐2‐phenylindole (DAPI; Vector Laboratories Inc.), and the cells were visualized using an IX73 fluorescent microscope (Olympus Optical Co., Ltd.), or an Axio Imager.a2 (Carl Zeiss AG.). The primary antibodies were mouse anti‐human OCT4 (SC‐5279) and Nanog (SC‐293121; both from Santa Cruz Biotechnology Inc.), Stage‐specific embryonic antigen 4 (SSEA‐4, MAB4304; Merck Millipore), rabbit anti‐human Ets variant transcription factor 2 (ETV2; ab181847), rabbit anti‐human CD31 (ab28364), rabbit anti‐human Runt‐related transcription factor 1 (RUNX1; ab35962), rabbit anti‐human FLK‐1 (KDR) (ab2349), mouse anti‐human CD71 (ab9179), rabbit anti‐human CD235a (ab129024), mouse anti‐human CD105 (ab230925), mouse anti‐human CD44 (ab254530), and mouse anti‐human SOX9 (ab76997; all from Abcam Plc.); mouse anti‐human Brachyury (14‐9770‐80) and mouse anti‐human CD11b (14‐0118‐82; both from eBioscience), goat anti‐human TIE2 (AF313, R&D Systems Inc.), Rabbit anti‐CD31 antibody (ab28364, Abcam) and Sheep anti‐Von Willebrand Factor (vWF) antibody (ab11713, Abcam) and mouse anti‐human CD34 (826401, BioLegend). Primary signals were detected using isotype‐matched IgG antibodies.

### 2.4 Wright‐Giemsa staining

Hemogenic ECs derived from PSCs were dropped onto microscope slides and air‐dried, then haematopoietic lineage cells were stained with Wright‐Giemsa Solution (ab245888, Abcam) as described by the manufacturer. Briefly, the cells were fixed in methanol for 5 min, then stained with Wright‐Giemsa solution for 5 min. The cells were washed with distilled water, rinsed with PBS, and covered with the mounting solution.

### 2.5 Colony‐forming unit assay and tube formation assay

We evaluated whether HE derived from PSC had the functions or potency of haematopoietic progenitors by incubating HE cells (2 × 10^4^) derived from PSCs in 500 μl of MethoCult™ medium (H4434, StemCell Technologies Inc.) for 14 days. For the tube formation assay, Reduced Growth Factor Basement Membrane Matrix, LDEV‐free Matrigel (250 μl, 354230, Corning) was added to the 24‐well plate and solidified by incubation at 37°C for 1 h. Subsequently, 2 × 10^5^ cells of CHA52‐ and H1‐derived HEs were plated onto each matrigel‐coated well with EGM‐2 media and then incubated at 37°C with 5% CO_2_ for 9 h. After removing the media, 4% PFA was added for fixation. The cells were visualized using a light microscope. The formation of colonies and tube‐like structures was assessed using a CKX53 microscope (Olympus).

### 2.6 Quantitative (q) RT‐PCR

Total RNA was extracted from human cells using TRIzol reagent (Ambion) as described by the manufacturer. cDNA was then synthesized using reverse transcriptase kits (RT200, Enzynomics). Fragments were amplified by qRT‐PCR using primers (Table 1), SYBR Green (RT500M, Enzynomics), and the CFX96™Real‐Time System (Bio‐Rad Laboratories Inc.). The relative mRNA expression of target genes was calculated using the comparative CT method. All target genes were normalized to *GAPDH* in multiplexed triplicate reactions. Differences in CT values were calculated for each target mRNA by subtracting the mean value of *GAPDH* (relative expression = 2^−ΔCT^).

**TABLE 1:** Primers and probes for quantitative RT‐PCR

Genes		Primers and probes (5′‐3′)
Human	Forward	GGTGGTCTCCTCTGACTTCAACA
*GAPDH*	Reverse	GTGGTCGTTGAGGGCAATG
Human	Forward	AAATCCTCTTCCTCTGAGGCTGGA
*CD34*	Reverse	AAGAGGCAGCTGGTGATAAGGGTT
Human	Forward	CTCAGCTCTCACCGTTTGCT
*ETV2*	Reverse	ATGGGACCTCGGTGGTTAGT
Human	Forward	CCTCTACTCCAGTAAACCTGATTGGG
*KDR*	Reverse	TGTTCCCAGCATTTCACACTATGG
Human	Forward	CTTCACAAACCCACCGCAAG
*RUNX1*	Reverse	GGCTGAGGGTTAAAGGCAGT
Human	Forward	TGTATTTCAAGACCTCTGTGCACTT
*CD31*	Reverse	TTAGCCTGAGGAATTGCTGTGTT
Human	Forward	CCTTGGTCACATCTTCACATCAC
*vWF*	Reverse	TCATTGGCTCCGTTCTCATCAC
Human	Forward	AATGCCCCGGAGTTTGC
*CDH5*	Reverse	TGGACAGCGTTCTCACACACTT
Human	Forward	CCTGCCTGACTGTGCTGTTG
*TIE2*	Reverse	TGCACATTTGCCCTCTTCAA

### 2.7 Statistical analysis

All results are presented as means ± SEM and were statistically analysed by Mann–Whitney *U* tests and Student *t*‐tests for between‐group comparisons using GraphPad Prism v.4 (GraphPad Software Inc). Values with *p* < 0.05 were considered statistically significant.

## 3 RESULTS

### 3.1 Human HE cells derived from hPSCs were CD34^dim^

**FIGURE 1:**
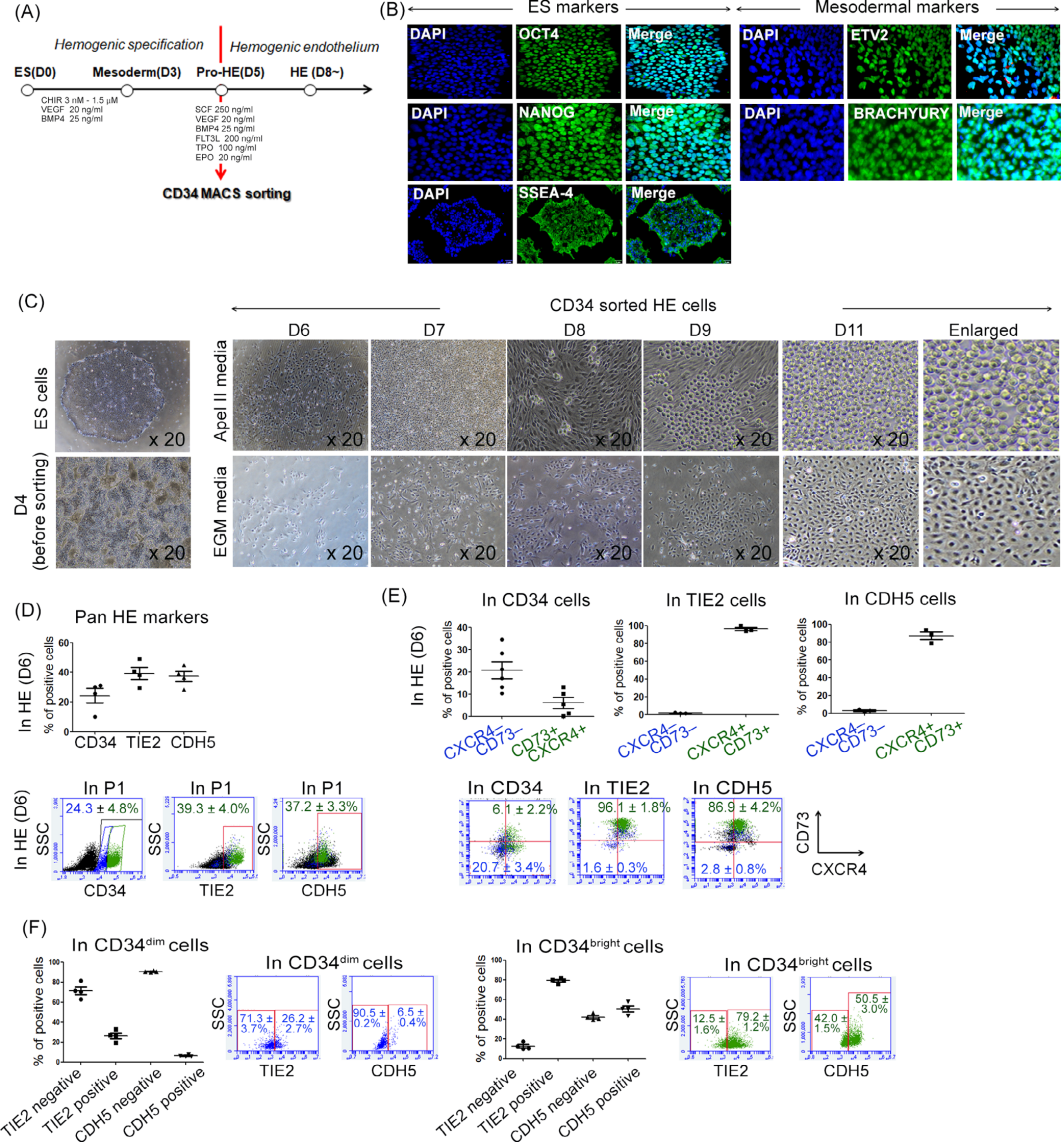
Bipotential hemogenic endothelium (HE) generated from pluripotent stem cells (PSCs) and conventional HE markers expressed on differentiation Day 6. (A) Schema for generating HE cells from PSCs via mesodermal lineages. (B) Immunocytochemistry findings of qualitatively expressed OCT4, NANOG and SSEA‐4 in PSCs and mesodermal markers ETV2 and *Brachyury* in mesodermal lineage cells. (C) Morphological changes indicate the bipotential of HE cells to differentiate into haematopoietic and endothelial lineages. Magnification 20×. (D) Expression of CD34, TIE2 and CDH5 markers in HE cells at Day 6 after differentiation. Cells were analysed using FACS analysis. Data are shown as means ± SEM of at least three experiments. (E) Proportions of undifferentiated CXCR4^−^CD73^−^ and differentiated CXCR4^+^CD73^+^ cells in HE expressing markers. CD34 cells contained the most CXCR4^−^CD73^−^ cells. Data are shown as means ± SEM from at least three experiments. (F) Unlike TIE2^+^ and CDH5^+^ cells, CD34^+^ cells comprised CD34^dim^ and CD34^bright^ types. High proportions of TIE2 and CDH5 were detected in CD34^bright^ and CD34^dim^ populations, and high proportions of CXCR4^−^CD73^−^ cells expressed low levels of TIE2 and CDH5. Data are shown as means ± SEM of at least three experiments.

We developed a protocol in which functional HE derived from hPSCs were cultured for 8 days in a medium supplemented with specific cytokines (Figure 1A). We applied MACS with CD34 beads to differentiated PSCs 2 days after the mesoderm stage on Day 5. We found that octamer‐binding transcription factor 4 (OCT4), homeobox protein NANOG, and SSEA‐4 were conventionally expressed in CHA52‐PSCs, and the mesodermal markers ETV2 and brachyury (T) were abundantly expressed in differentiated cells at Day 3, implying the onset of hPSC differentiation accompanied by loss of pluripotency (Figure 1B). After Day 3, the cells in the defined medium for HE specification were incubated in Matrigel®‐coated plates. We removed non‐HE cells without excessive aggregation on Day 5, and CD34^+^‐positive cells were selected by MACS to determine their enrichment in HE cell populations. Hemogenic ECs have the bipotential capacity to differentiate into ECs and haematopoietic lineage cells. Therefore, we incubated HE cells for 11 days in APEL™ 2 and EGM‐2 media that are respectively specific to blood lineage and ECs. Figure 1C shows that the cells specifically differentiated according to the medium. The CD34^+^ cells in APEL™ 2 medium produced blood lineage cells such as erythroblasts, whereas CD34^+^ cells in EGM‐2 based medium generated ECs with a cobblestone‐like appearance (Figure 1C). Similar to CHA52, markers for stem cell were clearly detected and differentiation into haematopoietic lineages via HE was confirmed in APEL™ 2 medium in H1 and H9 (Figure S1A–C). The conventional markers, CD34, TIE2 and CDH5 were expressed in HE cells at ratios of 24.3 ± 4.8%, 39.3 ± 4.0% and 37.2 ± 3.3%, respectively (Figure 1D). The CXCR4^−^CD73^−^ phenotype was obvious in HE on Day 6. Most TIE2^+^ and CDH5^+^ cells had the CXCR4^+^CD73^+^ phenotype, implying full differentiation. Meanwhile, the CD34^+^ cells had both the CXCR4^−^CD73^−^ (CD34, 20.7 ± 3.4%; CDH5, 2.8 ± 0.8%; TIE2, 1.6 ± 0.3%) and CXCR4^+^CD73^+^ (CD34, 6.1 ± 2.2%; CDH5, 86.9 ± 4.2%; TIE2, 96.1 ± 1.8%) phenotypes suggesting that undifferentiated cells persisted among the CD34^+^ HE cells on Day 6 (Figure 1E). The CD34^+^ population notably comprised dim (CD34^dim^) and bright (CD34^bright^) populations. Thus, we examined the relevance of HE markers. Figure 1F shows that TIE2^−^ and CDH5^−^ were enriched in the CD34^dim^ but not in the CD34^bright^ population, whereas TIE2^+^ and CDH5^+^ were more enriched in the CD34^bright^ than that in the CD34^dim^ population. These findings suggested that the CD34^bright^ population enriched in TIE2 and CDH5 markers function as differentiated ECs with the CXCR4^+^CD73^+^ phenotype. Although the CD34^dim^ population was mostly TIE2^−^ and CDH5^−^, CD34^dim^ cells seemed to have functional HE potential due to the enriched CXCR4^−^CD73^−^ phenotype (Figure 1E,F). Thus, we putatively considered that early CD34^dim^ HE cells function as definitive HE compared to those expressing other markers.

### 3.2 Haematopoietic lineage cells were induced and sustained by CXCR4^−^CD73 ^−^ HE CD34 cells preserved by BMP4

**FIGURE 2:**
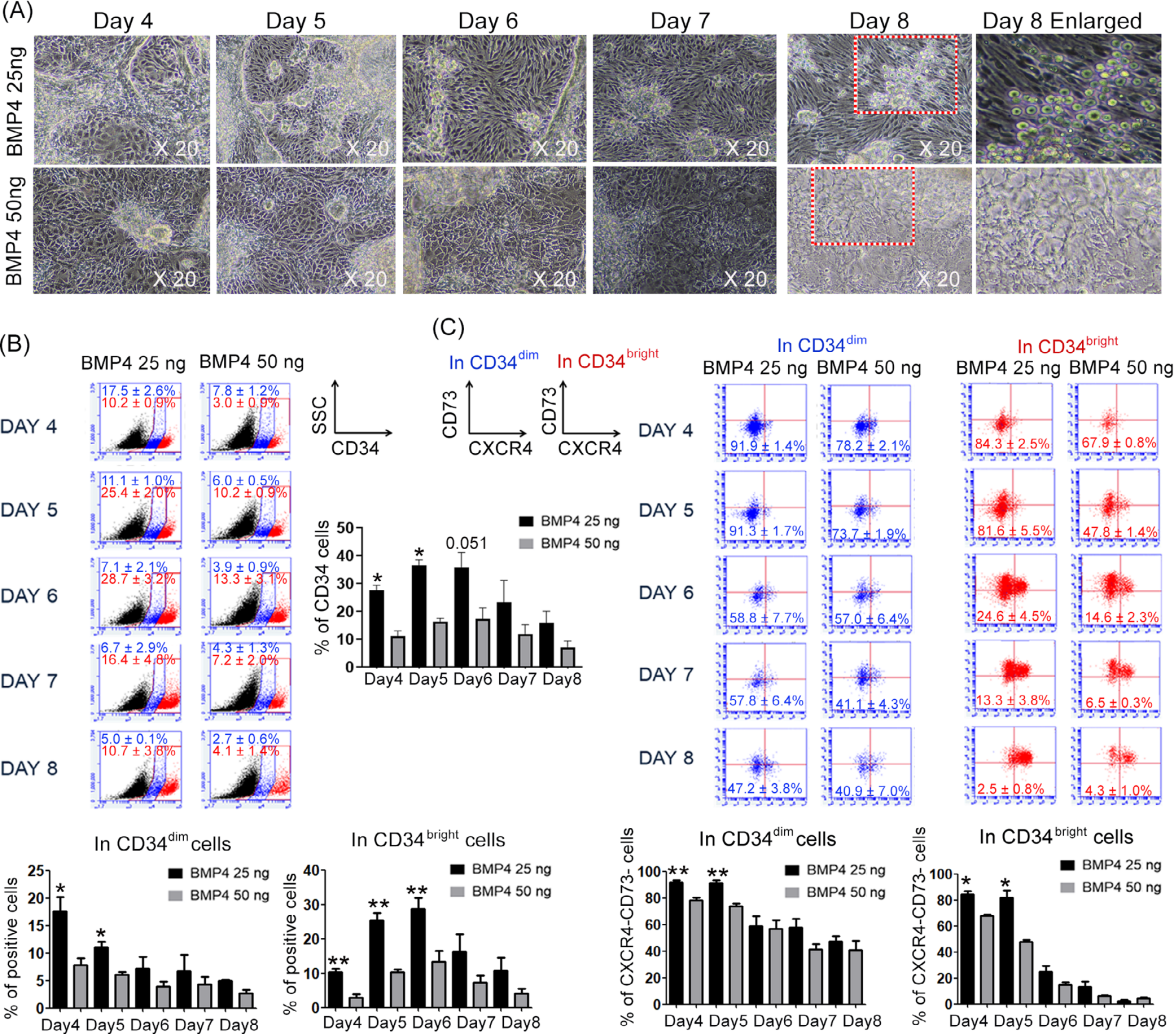
Bone morphogenetic protein 4 (BMP4) (25 ng/ml) induced hemogenic endothelium (HE) optimization from pluripotent stem cells (PSCs) and sustained definitive HE properties. (A) Representative morphology of PSC‐derived HE according to BMP4 concentrations. Magnification 20×. (B) Proportions of CD34^+^, CD34^dim^ and CD34^bright^ cells in differentiated HE cells according to BMP4 concentrations. Red and blue plots, CD34^bright^ and CD34^dim^ populations, respectively. Images are representative of three independent experiments (*p < 0.05; **p < 0.01; *n* = 3 per group). (C) Proportions of CXCR4^−^CD73^−^ cells in CD34^dim^ and CD34^bright^ cells based on BMP4 concentration. Red and blue plots, CD34^bright^ and CD34^dim^ populations, respectively. Representative images from three independent experiments (*p < 0.05; **p < 0.01; *n* = 3 per group).

We investigated whether early CD34^dim^ HE cells can function as bona fide HE. We analysed the CXCR4^−^CD73^−^ phenotype in CD34^dim^ and CD34^bright^ populations using FACS. We also cultured HE cells with BMP4 (25 and 50 ng/ml) under EHT conditions for 8 days to determine whether BMP4 sustains and preserves the functions of these cells. Among the HE population incubated with BMP4 (25 ng/ml), CD34^+^ cells emerged on Day 5, and their territory within HE was sustained without destruction. Erythroblasts budded from the HE cells on Day 8. Incubation with BMP4 (50 ng/ml) resulted in a decreased HE population, and the HE territory gradually disappeared. This ultimately resulted in the failure to robustly produce erythroblasts from HE (Figure 2A). The proportion of CD34^+^ cells was higher in HE cells incubated with 25 ng/ml of BMP4 than that with 50 ng/ml. The numbers of CD34^+^ cells incubated with BMP4 (25 ng/ml) were significantly increased on Days 4 and 5 (Figure 2B). The number of CD34^dim^ cells peaked on Day 4, then rapidly declined, indicating a reduction in the total cell population. Meanwhile, the numbers of CD34^bright^ cells increased until Day 6, then decreased from Day 7 regardless of the BMP4 concentration. Although the cultured cells instantly and rapidly differentiated due to unrestricted CD34^+^ isolated cells, the proportion of CD34 cells was higher after incubation with 25 ng/ml BMP4 than that with 50 ng/ml (Figure 2B). Undifferentiated cells with the CXCR4^−^CD73^−^ phenotype in the CD34^dim^ and CD34^bright^ populations were also incubated with both concentrations of BMP4. The overall frequency of CXCR4^−^CD73^−^ cells was maintained at >91.3% when functional HE CD34^dim^ cells were incubated with BMP4 (25 ng/ml) and persisted until Day 5. This implied that BMP4 (25 ng/ml) protected the HE territory against rapid differentiation. On Day 5 of differentiation, tentatively designated as the optimal time to isolate HE, the proportion (%) of CXCR4^−^CD73^−^ in CD34^dim^ cells incubated with BMP4 (25 ng/ml) was 91.3 ± 1.7%, whereas that of CXCR4^−^CD73^−^ in CD34^bright^ cells incubated with BMP4 (50 ng/ml) was 73.7 ± 1.9%. This remarkably decreased after 5 days regardless of the BMP4 concentration. Thus, we selected 25 ng/ml as the optimal concentration of BMP4 required for functional HE development (Figure 2C). The CD34^bright^ cells could not sustain the CXCR4^−^CD73^−^ phenotype compared with the CD34^dim^ population. We then compared the coating materials, vitronectin and Matrigel®, and induced PSC differentiation into the mesodermal lineage using CHIR99021, a GSK3β inhibitor that mimics the Wnt signal activation due to specific targeting capability.^28^, ^29^ PSCs were seeded onto culture dishes and incubated with 3 μM or 3 nM CHIR99021 for 3 days to induce mesodermal lineages. Matrigel™ facilitated differentiation into the mesodermal lineage more effectively than vitronectin (data not shown). Hemogenic ECs emerged after incubation with 3 nM CHIR99021 for 3 days, whereas 3 μM CHIR99021 did not generate any blood lineages in CHA52 ESCs (Figure S2A). Flow cytometry confirmed that the overall proportions (%) of HE markers, TIE2, CD34 and CDH5 were higher when ESCs were incubated with 3 nM than that with 3 μM of CHIR99021 (Figure S2B). Meanwhile, it was found that the HE generation in H1 and H9 ESCs was rather increased at the CHIR99021 1.5 μM, but not 3 nM. In despite the difference in ESC cell lines according to the CHIR99021 concentration that induces CD34^+^ HE, we further performed FACS analysis to investigate whether the phenotype of definitive HE, CD34^dim/bright^ was enriched at Day 5.

### 3.3 CD34^dim^ ^/bright^ cells contained the most CXCR4^−^CD73 ^−^ cells on Day 5

**FIGURE 3:**
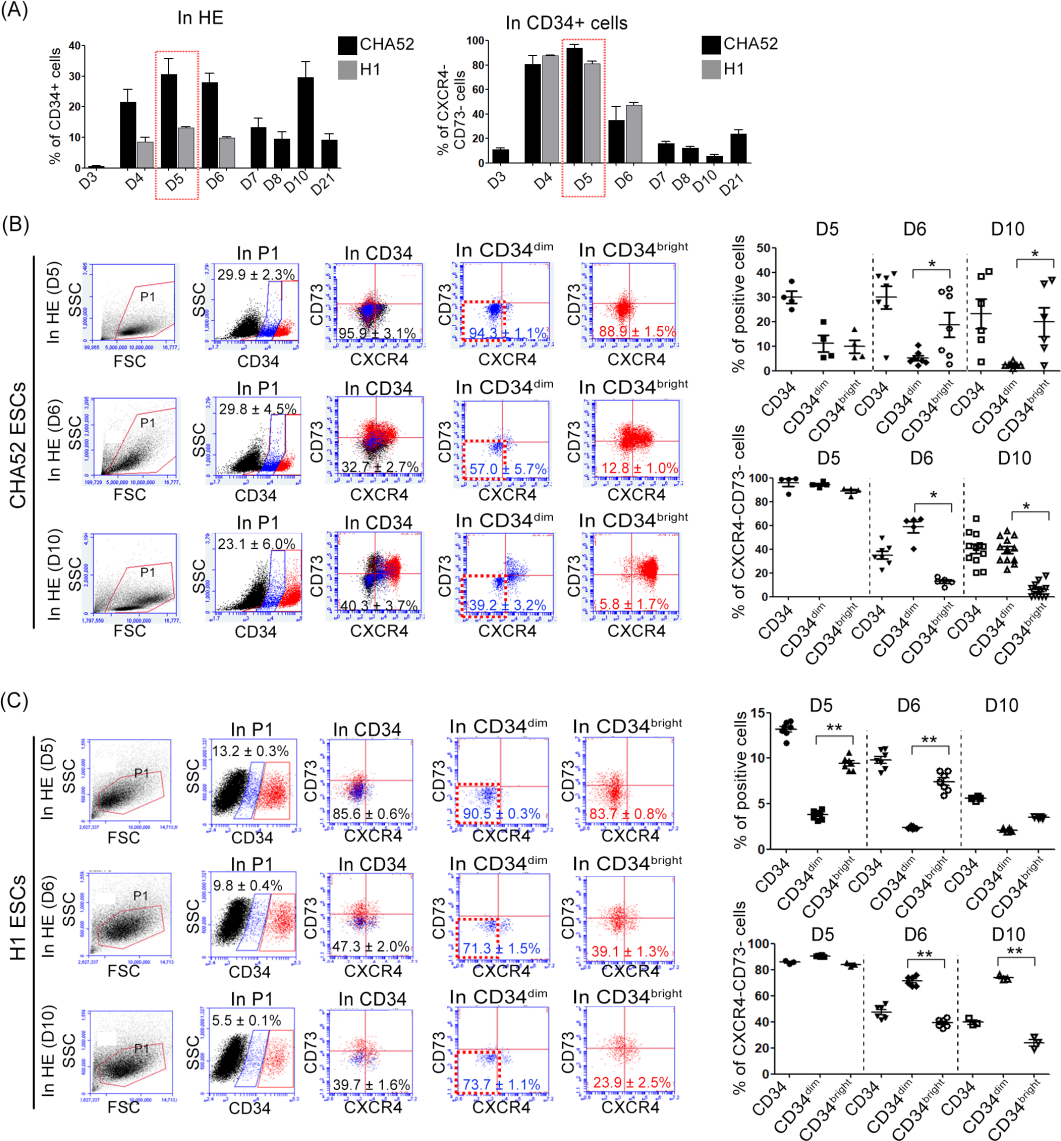
Flow cytometry of hemogenic endothelium (HE) over time using differentiated HE. (A) Optimal day for magnetic‐activated cell sorting sorting was when proportions of CXCR4^−^CD73^−^ cells among CD34 cells from CHA52 and H1 ESCs were maximal. CD34^+^ and CD34^dim/bright^ cells on Day 5 contained the most CXCR4^−^CD73^−^ cells. Thus, Day 5 was taken as the optimal time to acquire purified HE. Red box, maximal proportions of CD34 cells and CXCR4^−^CD73^−^ among CD34^+^ cells. (B) Day 5, 6 and 10 were measured the percentage of CXCR4^−^CD73^−^ cells in CD34 cells from CHA52 ESCs. CD34^bright^ cells differentiated more rapidly than CD34^dim^ cells over time, results in rapidly reduction of CXCR4^−^CD73^−^ cells. Data are from at least three independent experiments (*n* = 4–16 per group). Red and blue plots, CD34^bright^ and CD34^dim^ populations, respectively. (C) Day 5, 6 and 10 were measured the percentage of CXCR4^−^CD73^−^ cells in CD34 cells from H1 ESCs. CD34^bright^ cells differentiated more rapidly than CD34^dim^ cells over time, results in rapidly reduction of CXCR4^−^CD73^−^ cells. Data are from at least three independent experiments (*n* = 3–7). Red and blue plots, CD34^bright^ and CD34^dim^ populations, respectively. *p < 0.05, **p < 0.01

Based on the results shown in Figure 2, we further investigated the proportions (%) of CXCR4^−^CD73^−^ cells among CD34^+^ HE from PSCs incubated with 25 ng/ml BMP4 for 3 to 21 days. The proportions (%) of CD34^+^ and CXCR4^−^CD73^−^ cells among CD34^dim/bright^ HE cells from CHA52 and H1 were maximal, respectively, on Day 5 (Figure 3A). In contrast, rapid differentiation on Day 6 was indicated by a significant increase and decrease in CXCR4^+^CD73^+^ and CXCR4^−^CD73^−^ cells, respectively, regardless of the CD34^dim^ or CD34^bright^ fraction. By Day 10, the number of CXCR4^−^CD73^−^ cells continuously decreased in CD34^+^ cells among CD34^dim^ and CD34^bright^ HE cells (Day 6: 57 ± 5.7% and 12.8 ± 1.0%, respectively; Day 10, 39.2 ± 3.2% and 5.8 ± 1.7%, respectively; Figure 3B). To address H1 and H9 ESCs, we designed BMP4 and CHIR concentration serially (Figure S3A) to optimize HE and found that H1‐derived CD34^+^ HE was the highest peaked in BMP4 25 ng/ml with CHIR 1.5 μM. Since more than 15% of cells are required to isolate MACS sorting, we intentionally excluded the culture condition for the low percentage of CD34^+^ HE and selected the culture condition marked Group 3 (BMP4 25 ng/ml with CHIR 1.5 μM) for H1 and H9 for further experiment (Figure S3B). In case of H1, the percentage of CD34^+^ HE was decreased with 2.2‐fold, compared to that of CHA52‐derived CD34^+^ HE, but CXCR4^−^CD73^−^ cells in CD34^+^ HE still possess the highest frequency at Day 5 (in CD34^+^ HE, CHA52, 29.9 ± 2.3%, H1, 13.2 ± 0.3%; in CXCR4^−^CD73^−^ cells in CD34^dim^ HE, CHA52, 94.3 ± 1.1%, H1, 90.5 ± 0.3%). Similar to CHA52, the proportion of definitive HE was rapidly decreased in a day‐dependent manner. Even low number of H1‐derived CD34^+^ HE, reproducibility of the culture condition expecting a high yield of HE at Day 5 was confirmed (Figure 3C and Figure S3C). Although H9 cell lines failed to display high CD34^+^ HE, the CXCR4^−^CD73^−^ cells in CD34^dim^ HE were still maintained with the highest at Day 5, suggesting consistency for prospectively isolating HE according to present protocol (Figure S3D).

### 3.4 CD34 HE was simultaneously displayed a haematopoietic and endothelial properties

**FIGURE 4:**
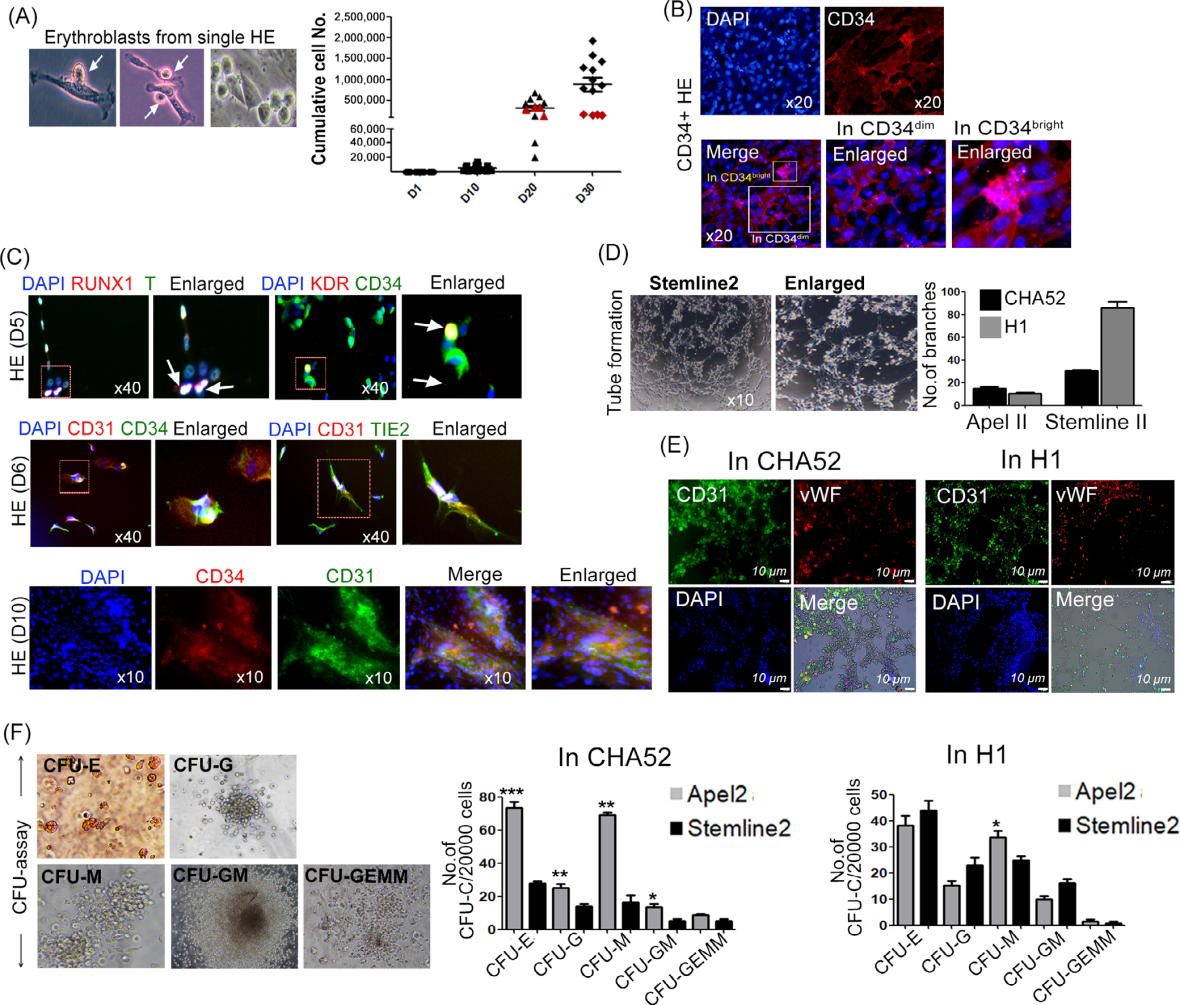
The bipotential capability of hemogenic endothelium (HE) was evident in CD34^+^ purified HE. (A) Endothelial‐to‐haematopoietic transition (EHT) from single HE and HE proliferation with erythroblasts generated in HE. White arrows, EHT from HE. Cumulative cell numbers show robust HE expansion for up to 20 days after differentiation. HE proliferation was arrested by Day 30, suggesting that the cell growth gradually decreases after 20 days. Red dots, HE cells with arrested growth. Data are derived from at least three independent experiments (*n* = 13–18 per group). (B) Immunocytochemistry findings show CD34^dim^ and CD34^bright^ cells in pluripotent stem cell‐derived HE. CD34^dim^ cells remained adherent, whereas CD34^bright^ cells differentiated into haematopoietic lineages. Magnification, 20×. (C) Immunochemistry findings of RUNX1, Brachyury (T), KDR and CD34 expression in early HE on Day 5. From Day 6, CD31, CD34 and TIE2 were co‐expressed in HE and abundantly expressed over time. Magnification 40×, 10×. (D) Representative images of tube formation assay in CHA52 and H1 ESCs. The hESC‐derived CD34^+^ HEs were cultured in Matrigel‐coated 24‐well plates with EGM media for 9 h. The ability of HE to differentiate into endothelial lineages determined by tube formation in early HE. Stemline® 2‐based media was proper to encourage endothelial lineages. (E) Immunocytochemistry results showed endothelial properties in tube formed HE from hESCs. CD31 (green), Von Willebrand Factor (red), and nuclei stained with DAPI (blue). Scale bar: 10 μm. (F) The ability of HE to differentiate into haematopoietic lineages determined by CFU assays in early HE. CFU‐colonies, especially CFU‐M, ‐G and ‐E was significantly increased in APEL™ 2‐based media, compared to that of Stemline® 2‐based media. Data are shown as means ± SEM from at least three independent experiments (*n* = 7–13 per group; *p < 0.05; **p < 0.01; ***p < 0.001).

These HE cells had the normal rod‐like endothelial morphology with budding erythroblasts, and the number of accumulated cells significantly increased between 10 and 20 days of HE incubation. Cells with arrested proliferation emerged after Day 20 (Figure 4A), suggesting rapid HE growth as in embryogenesis. Our data showed that CXCR4^−^CD73^−^ undifferentiated HE CD34 cells were restricted during Day 5. Therefore, we regarded Day 5 as the optimal time to acquire purified HE. We further examined the prevalence of CD34^dim^ or CD34^bright^ cells using immunocytochemistry. We observed that the morphology of CD34^bright^ cells had already changed (EHT phenomenon), and CD34^dim^ cells persisted as adherent HE cells (Figure 4B). The expression of EHT, KDR, T and RUNX1 was detected in early HE. Additionally, CD31, TIE2 and CD34 were co‐expressed in stable HE cells on Days 6 and 10 (Figure 4C). We investigated the biopotential of HE by counting colony‐forming unit (CFU) and the formation of tubes. In both cells lines, HEs can make tube formation with expression of marker for ECs such as CD31 and vWF. These HE cells still generated haematopoietic lineage cells expressing EC markers, suggesting bipotent capability of CD34 HE (Figure 4D). Also, PCR data also displayed that EC markers including *CD31*, *vWF*, *CDH5*, *TIE2* and *ETV2* were significantly increased in CD34 sorted cells (Figure S4A) and matured ECs with tube formation without haematopoietic lineage cells were clearly detected in EGM medium at Day 35 (Figure S4B), suggesting the endothelial characteristics of CD34^+^ HE. The potential for ECs was enhanced by Stemline® II with EC differentiation, compared to that of APEL™ 2 media. In contrast, CHA52‐ and H1‐derived CFU‐colonies comprising CFU‐G, E, M, GM, and granulocyte, erythrocyte, monocyte, megakaryocyte were significantly increased in APEL™ 2 media (Figure 4F). The bipotent capability of CD34^+^ HE cells were confirmed by capacity for the CFU‐colonies and tube formation implied EC function.

### 3.5 Incubating purified HE for 5 days with BMP4 and CHIR99021 generated haematopoietic lineage cells

**FIGURE 5:**
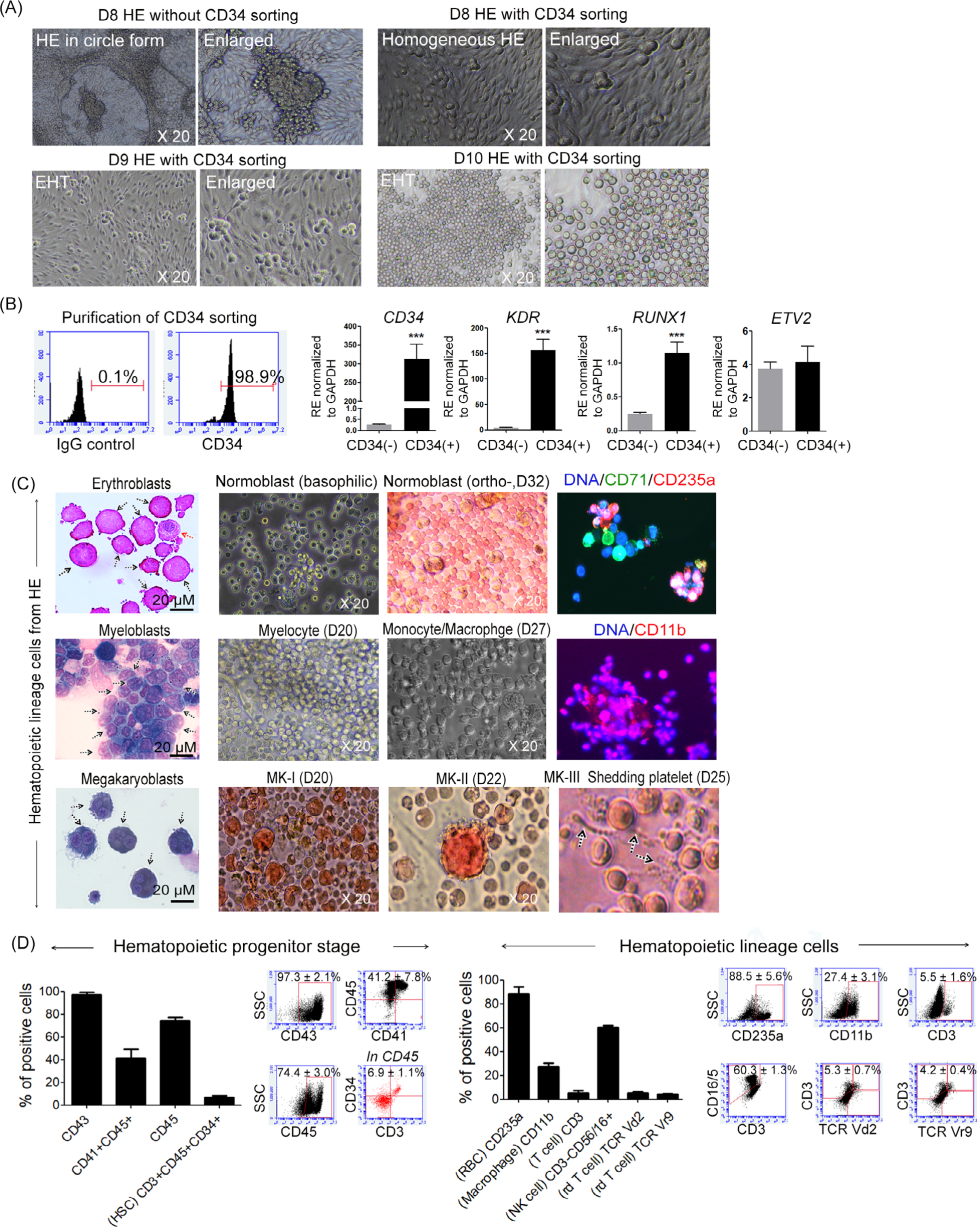
Purified hemogenic endothelium (HE) can differentiate into all haematopoietic lineage cells. (A) Cell morphology in HE before and after CD34 sorting. Purified and homogenous HE underwent EHT on Day 8 after sorting. Magnification, 20×. (B) CD34 purification was confirmed and mRNA expression of representative haematopoietic lineage committed factors in CD34^+^ and CD34^−^ cells measured by qRT‐PCR. (C) Giemsa staining in differentiated cells from purified HE. Scale bar, 20 μM. All haematopoietic lineage cells were generated from CD34‐sorted HE within 32 days. Lineage cell differentiation from HE was confirmed using specific lineage markers. Black arrows, shedding platelets, MK‐III types. Magnification, 20×. (D) Flow cytometry data show abundant haematopoietic progenitors, implying full commitment toward blood lineages. CD45^+^CD34^+^CD3^+^ cells also emerged from purified HE from Day 10 (*n* = 4–6). Markers of maturation also increased in differentiated cells by Day 40. Data are derived from at least two independent experiments with duplicates (*n* = 4–7). ***p < 0.001

We established an efficient system for differentiating HE from PSCs using CHIR99021 and BMP4, and then sorting the cells with CD34. We confirmed that Day 5 was the optimal time for isolating the maximal numbers of CXCR4^−^CD73^−^ cells in CD34^dim/bright^ populations of HE cells. We then purified the HE cells using MACS sorting with a CD34 antibody. The purified PSC‐CD34^+^ cells robustly generated morphologically homogeneous erythroblasts from Day 8 via the EHT for 30 days (Figure 5A). However, the EHT of CD34^+^ HE cells was lower on Days 3 and 6 after seeding compared to that on Day 5 (data not shown). The cells before sorting were morphologically differentiated into an HE population and hepatic‐like cells. These mixed and dynamic cells rapidly aggregated with three‐dimensional architecture. The hepatic‐like cells dominated the heterogeneous cells and ultimately expanded, resulting in a loss of HE territory. The hepatic‐like cells also clustered together like blood cells and emerged as in the EHT. However, these aggregates did not float like erythroblasts. Most hepatic‐like cells were produced when PSCs did not commit to mesodermal differentiation, which led to a sustained PSC colony. These cells ultimately generated into hepatocytes and formed a monolayer of polygonal cells (Figure S5A). These cells did not express CDH5, CD43 and CD45, which are markers of pan haematopoietic cells regardless of being adherent or suspended. The MSC markers, CD105, CD44 and CD90, were partly or abundantly expressed in adherent cells; however, the hepatocyte marker SOX9 was abundantly expressed in adherent cells, indicating hepatocyte differentiation (Figure S5B,C). However, CD34^+^ sorted HE cells on Day 5 were highly homogeneous and >98.9% pure according to their phenotype. We measured the expression of transcription factors for definitive HE to determine the haematopoietic activity of the sorted CD34^+^ and CD34^−^ cells. The results of qRT‐PCR revealed significantly more abundant mRNA expression of *KDR*, and *RUNX1* in the CD34^+^ than in the CHA52‐, H1, and H9‐derived CD34^−^ cells (Figure 5B and Figure S6). These HE cells underwent an extreme EHT (Videos S1 and S2). We investigated whether all blood lineage cells were generated from CD34‐sorted HE by incubating them in conditioned media for 35 days. Figure 4B shows that erythroblasts emerged during the early stage, followed by erythroblasts, myeloblasts and megakaryoblasts. We detected CD71 and CD235a in orthochromatic normoblasts on Day 32, and macrophages expressing CD11b were induced from HE cells on Day 27. Megakaryoblasts (MK‐I) were identified on Day 20 and further developed into MK‐II and MK‐III, leading to shedding platelets on Day 25 (Figure 5C and Video S3). We analysed the lineages of cells harvested between 25 and 40 days after differentiation from HE using FACS to determine whether lymphoid lineage cells emerged from bona fide CD34‐sorted HE cells. Most cells expressed CD43 (97.3 ± 2.1%), a marker of blood lineage commitments, CD41 (41.2 ± 7.8%), a marker of haematopoietic progenitor specification, and CD45 (74.4 ± 3.0%), a pan haematopoietic cell marker, by Day 30. Our culture system using purified HE revealed that the proportion (%) of HSC progenitor CD3^+^CD34^+^ in CD45^+^ cells on Day 21 was 6.9 ± 1.1%.^7^, ^15^ These findings suggested that bona fide HE cells were generated from PSCs by optimizing the sorting method. The ratios of haematopoietic lineage cells determined by measuring CD235a (88.5 ± 5.6%), CD11b (27.4 ± 3.1%), and CD3 (5.5 ± 1.6%) for lymphoid lineages, CD56/16^+^CD3^−^ natural killer (NK) cells (60.3 ± 1.3%), and TCR γδ T cells (5.3 ± 0.7%) among CD45^+^ cells indicated that these haematopoietic lineage cells were stably generated from CD34‐sorted HE cells (CD3, 5.5 ± 1.6%; CD56/16^+^CD3^−^, 60.3 ± 1.3%; TCR Vδ2^+^CD3^+^, 5.3 ± 0.7%; TCR Vγ9^+^CD3^+^, 4.2 ± 0.4%). These findings indicated that CD34‐sorted HE could function as bona fide HE that can generate both lymphoid lineage cells and HSCs (Figure 5D).

Collectively, these data revealed that the CD34^dim^ population is a marker of functional HE and that bona fide HE could be acquired by simply purifying the CXCR4^−^CD73^−^‐enriched CD34^+^ population.

## 4 DISCUSSION

HE is an established pivotal reservoir where blood lineages are generated from PSCs. However, a prospective method of purifying HE cells has remained challenging. Homogeneous populations are challenging to completely purify from PSCs due to their diversity and dynamic properties. We overcame the caveats to haematological cell therapy by developing a simple method of HE purification. Because the crucial and inevitable role of HE is pivotal to achieving the functional conversion of haematopoietic lineage cells from PSCs, our method of HE purification should be prospectively assessed to access clinical trials. A primitive haematopoietic wave during PSC differentiation is considered the initial program of embryonic haematopoiesis; it is involved in myeloid lineage cells, including macrophages and erythroblasts, but not lymphoid lineage cells and HSCs. This process arises from the yolk sac in the blood islands.^30^ A second wave at a later stage of definitive haematopoiesis induces functional multipotent progenitors with lymphoid potential and HSCs.^31^, ^32^, ^33^ Most of these findings have been determined in mouse embryos. Nonetheless, embryonic‐derived haematopoietic processes in humans are controversial because they differ from mice in terms of development. That HSCs and lymphoid progenitors of definitive haematopoiesis are still considered products of definitive haematopoiesis and AGM, especially in the dorsal aorta, has reached consensus.^33^ Regardless of the controversy over the exact ancestor of human haematopoietic lineage cells, we demonstrated that HE differentiated using a defined medium could function as a key reservoir for haematopoietic lineage cells derived from PSCs.^16^, ^34^ Notch and BMP4 signalling specify arterial‐type definitive HE. We developed mesodermal lineage cells using CHIR99021 and BMP4, which stimulated the Notch signal pathway to fully commit to the mesoderm lineage.^10^, ^35^ Because HE is transient under conditions that might not allow long‐term culture and a progenitor reservoir, the appropriate conditions for HE culture were maintained without the induction of spontaneous differentiation by low concentrations of BMP4 and CHIR99021. Because the HE area was reduced due to more aggressive ES cells with the ability to overgrow, a prerequisite for HE generation from PSCs is that mesodermal differentiation must be completed without ES colony occupancy, and a reduction in liver‐like cells must be maintained. This phenomenon such as existence of ESC colonies was strongly decreased, when matrigel coating applied, leads to successful differentiate into haematopoietic lineages (Figure S7). When the purified HE population mainly occupied a territory, the ability to produce haematopoietic lineage cells was expanded compared to that of the sorted non‐purified HE cells. Although heterogeneous cells, including HE, were mixed 2 days after the mesodermal stage, relatively reproducible data were maintained using monotonous parameters such as single cells, non‐embryonic bodies, fewer cytokines, serum‐free media, and non‐stromal conditions. The clinical application of PSC‐derived lineage cells would require at least system reproducibility, simplicity, cost‐effectiveness, and be xenogeneic‐free.

We here developed and purified fully defined, enriched hPSC‐HE cells that are clinically compatible using a simple method. These differentiated HE cells with specification might serve as an early platform in vitro to generate myeloid and lymphoid lineage cells. Our purified HE could also serve as a novel platform for cell‐based therapy, especially haematopoietic lineage cells, as well as mechanistic studies in regenerative medicine and cancer biology.

## AUTHOR CONTRIBUTIONS

**Soo‐Been Jeon**: performed the experiments, analysed data; **A‐Reum Han**: performed the experiments, conducted FACS analysis; **Sunghun Lee**: performed the experiments, drafted the material and methods in manuscript; **Seung Chan Lee** and **Min Ji Lee**: performed the experiments, contributed to the analysis of data; **Soon‐Jung Park** and **Sung‐Hwan Moon**: performed the experiments, drafted the material and methods in manuscript; **Ji Yoon Lee**: conceived, designed and supervised the study, analysed data, drafted the manuscript.

## FUNDING INFORMATION

This work was supported by National Research Foundation of Korea (NRF) funded by the Korea government (MSIT) (No. 2021R1A2C1004571 and No. 2022R1A2C1006622).

## CONFLICT OF INTEREST

The authors declared no potential conflicts of interest.

## Supporting information


**FIGURE S1.** The generation of haematopoietic lineage cells from H1‐ and H9‐ ESCs was occurred via CD34^+^ HE by APEL™ 2‐ supplemented with optimized culture condition. (A) Immunochemistry findings of OCT4 (nucleus), NANOG (nucleus), and SSEA‐4 (cytoplasm) expression in ES colony, suggesting usage of confirmed PSCs. (B) Haematopoietic lineage cells were generated in sorted CD34 at Day 5. Magnification 10×.
**FIGURE S2.** HE from CHA52 ESCs was induced by low concentration of CHIR99021 (3 nM), but not high concentration (3 μM) and successfully generated haematopoietic lineage cells. (A) The representative morphology of PSC‐derived HE according to the concentration of CHIR99021. Magnification, 20×. (B) Proportions of TIE2, CD34 and CDH5 was examined using differentiated HE cells by CHIR. Red coloured plot indicates CXCR‐CD73‐ cells in CD34^dim^.
**FIGURE S3.** Optimization data for BMP4 (25, 50, 100 ng) and CHIR99021 (3 nM, 1 μM, 1.5 μM and 3 μM) in H1 and H9 ESC‐derived HE. (A) The eight groups were designed to select optimization culture condition for definitive HE. Selecting low CHIR99021 (3 nM) for CHA52 ESCs and high CHIR99021 (1.5 μM) for H1 and H9 ESCs was optimal for inducing definitive HE via mesodermal commitment, respectively. (B) FACS data showed that CHIR99021 1.5 μM and BMP4 25 ng/ml were required to generate high CD34^+^ cells in H1 ESCs, compared to that of other groups. Data are shown as means ± SEM of at least two experiments. (C) Flow cytometry data showed that H1 ESC‐derived CD34^+^ cells as well as CXCR4^+^CD73^+^ cells in CD34^+^ HE displayed the highest their frequency under Group 3. Data are shown as means ± SEM of at least three experiments. (D) Flow cytometry data showed that H9 ESC‐derived CD34^+^ cells as well as CXCR4^+^CD73^+^ cells in CD34^+^ HE displayed the highest their frequency under Group 3. Data are shown as means ± SEM of at least three experiments.
**FIGURE S4.** The ability of CD34^+^ HE was assessed for endothelial properties by PCR and tube formation. (A) After CD34 purification, qRT‐PCR analysis of mRNA expression related to endothelial cells was carried out using hESC‐derived CD34^+^ HEs. Data were normalized by glyceraldehyde‐3‐phosphate dehydrogenase (GAPDH) and expressed relatively to undifferentiated hESCs. Data are shown as means ± SEM from at least three independent experiments. (*n* = 3) (***p < 0.001). (B) The ability of CD34^+^ HE to differentiate into endothelial cells was determined by further culturing under EGM‐2 medium. These ECs formed tube immediately.
**FIGURE S5.** Failed HE from PSCs differentiated into hepatic like cells, which hampered HE territory maintenance. (A) The morphology of PSC‐derived hepatic like cells was shown. These cells further differentiated into hepatocytes with formation of monolayer polygonal shaped cells. Red box includes hepatocyte. Magnification, 20×. (B) Proportions of CD43, CDH5 and CD45 was highly reduced, suggesting non‐HE. (C) SOX9 was strongly expressed in adherent cells at Day 15 after differentiation, implying generation of hepatocyte.
**FIGURE S6.** A transcript level of representative haematopoietic lineage committed factors in CD34^+^ and CD34^−^ cells was measured by qRT‐PCR. Both H1 and H9 ESCs displayed the high level of genes including CD34, KDR, RUNX1 and ETV2 in CD34+ HE. Data are shown as means ± SEM from at least three independent experiments (**p < 0.01; ***p < 0.001).
**FIGURE S7.** Comparison data for matrigel and vitronectin coating in differentiation into haematopoietic lineage cells from PSCs. Morphological changes indicate that matrigel is better than vitronectin to differentiate into haematopoietic lineages. Vitronectin coated culture system failed to differentiation to haematopoietic lineage cells due to hampering of remained ESCs into haematopoietic lineages. Red circle, ESC colony. Magnification 10×.

**VIDEO S1.** The endothelial‐to‐hematopoieitc transition (EHT) from definitive hemogenic endothelium at early stage.

**VIDEO S2.** Erythropoiesis by the endothelial‐to‐hematopoieitc transition (EHT) after Day 12.

**VIDEO S3.** The development of megakaryblasts (MK‐I) into MK‐II and MK‐III, leading to shedding platelets

## Data Availability

The data that support the findings of this study are available from the corresponding author upon reasonable request.
