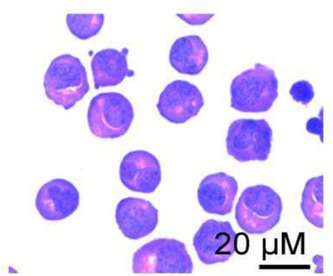# Correction to “CD34dim Cells Identified as Pluripotent Stem Cell‐Derived Definitive Hemogenic Endothelium Purified Using Bone Morphogenetic Protein 4”

**DOI:** 10.1111/cpr.70105

**Published:** 2025-09-07

S.‐B. Jeon, A.‐R. Han, S. Lee, S. C. Lee, M. J. Lee, S.‐J. Park, S.‐H. Moon, and J. Y. Lee, “CD34dim Cells Identified as Pluripotent Stem Cell‐Derived Definitive Hemogenic Endothelium Purified Using Bone Morphogenetic Protein 4,” *Cell Proliferation* 56, no. 2 (2023): e13366, https://doi.org/10.1111/cpr.13366.

The image depicting erythroblasts in Figure 5C was incorrect and was inadvertently duplicated in two related articles by the same author group. The correct image, corresponding to the erythroblasts used in the separation of hemogenic endothelium cells experiment, is shown below. The authors confirm that all experimental results and corresponding conclusions presented in the paper remain entirely unaffected. The authors sincerely apologise for this error.

**Corrected Figure 5C:**