# Supplementary material for: CD34dim cells identified as pluripotent stem cell‐derived definitive hemogenic endothelium purified using bone morphogenetic protein 4
Source: Cell Prolif. 2022 Dec 7;56(2):e13366. doi: 10.1111/cpr.13366 (PMC9890535; doi:10.1111/cpr.13366)
Supplement: Supplementary file 1 — FIGURE S1. The generation of haematopoietic lineage cells from H1‐ and H9‐ ESCs was occurred via CD34+ HE by APEL™ 2‐ supplemented with optimized culture condition. (A) Immunochemistry findings of OCT4 (nucleus), NANOG (nucleus), and SSEA‐4 (cytoplasm) expression in ES colony, suggesting usage of confirmed PSCs. (B) Haematopoietic lineage cells were generated in sorted CD34 at Day 5. Magnification 10×. FIGURE S2. HE from CHA52 ESCs was induced by low concentration of CHIR99021 (3 nM), but not high concentration (3 μM) and successfully generated haematopoietic lineage cells. (A) The representative morphology of PSC‐derived HE according to the concentration of CHIR99021. Magnification, 20×. (B) Proportions of TIE2, CD34 and CDH5 was examined using differentiated HE cells by CHIR. Red coloured plot indicates CXCR‐CD73‐ cells in CD34dim. FIGURE S3. Optimization data for BMP4 (25, 50, 100 ng) and CHIR99021 (3 nM, 1 μM, 1.5 μM and 3 μM) in H1 and H9 ESC‐derived HE. (A) The eight groups were designed to select optimization culture condition for definitive HE. Selecting low CHIR99021 (3 nM) for CHA52 ESCs and high CHIR99021 (1.5 μM) for H1 and H9 ESCs was optimal for inducing definitive HE via mesodermal commitment, respectively. (B) FACS data showed that CHIR99021 1.5 μM and BMP4 25 ng/ml were required to generate high CD34+ cells in H1 ESCs, compared to that of other groups. Data are shown as means ± SEM of at least two experiments. (C) Flow cytometry data showed that H1 ESC‐derived CD34+ cells as well as CXCR4+CD73+ cells in CD34+ HE displayed the highest their frequency under Group 3. Data are shown as means ± SEM of at least three experiments. (D) Flow cytometry data showed that H9 ESC‐derived CD34+ cells as well as CXCR4+CD73+ cells in CD34+ HE displayed the highest their frequency under Group 3. Data are shown as means ± SEM of at least three experiments. FIGURE S4. The ability of CD34+ HE was assessed for endothelial properties by PCR and tube formation. (A) After C [file CPR-56-e13366-s001.docx]

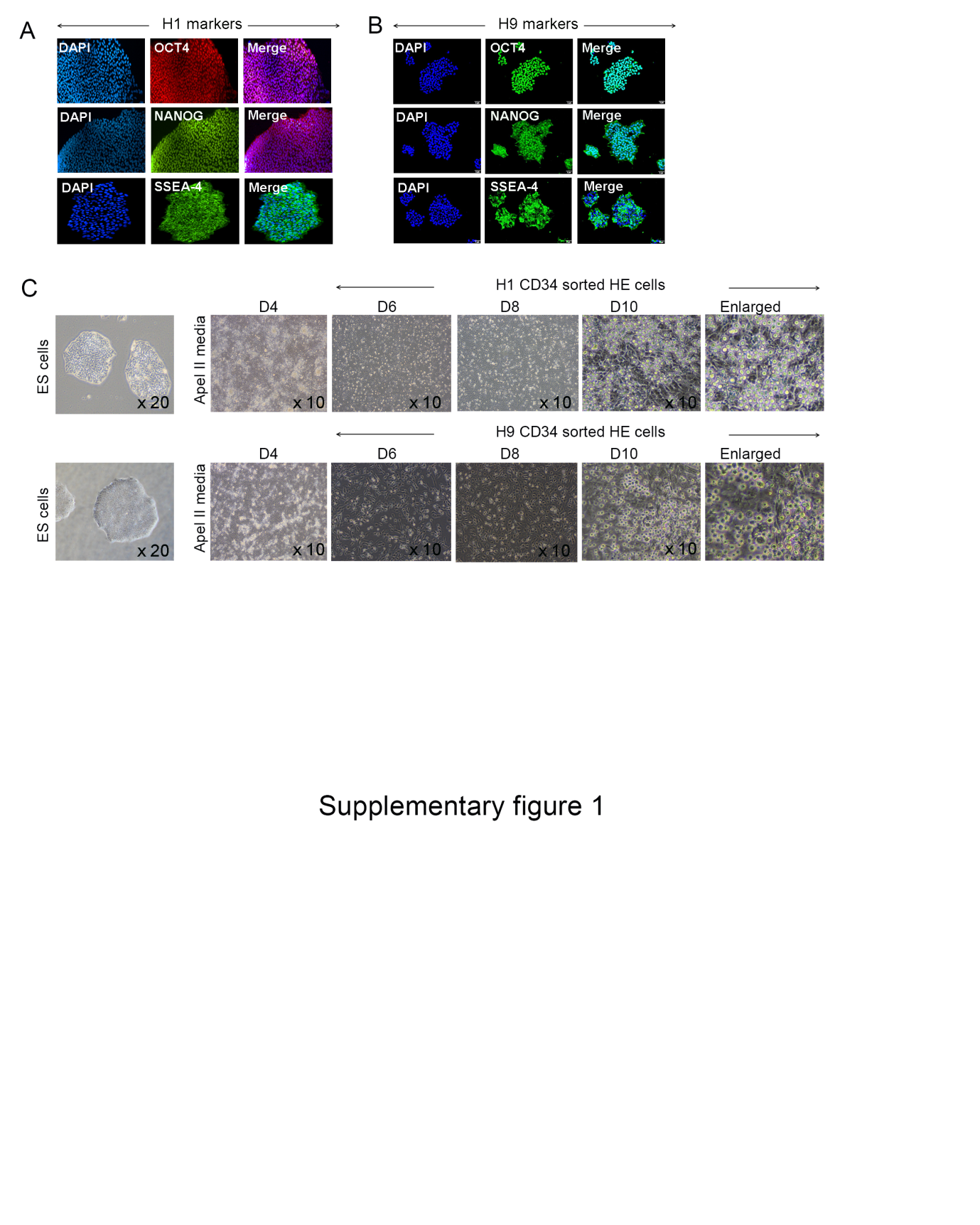


**Supplementary figure 1.** The generation of hematopoietic lineage cells from H1- and H9- ESCs was occurred via CD34^+^ HE by APEL^TM^ 2- supplemented with optimized culture condition. **(A)** Immunochemistry findings of OCT4 (nucleus), NANOG (nucleus), and SSEA-4 (cytoplasm) expression in ES colony, suggesting usage of confirmed PSCs. **(B)** hematopoietic lineage cells were generated in sorted CD34 at day 5. Magnification 10×.


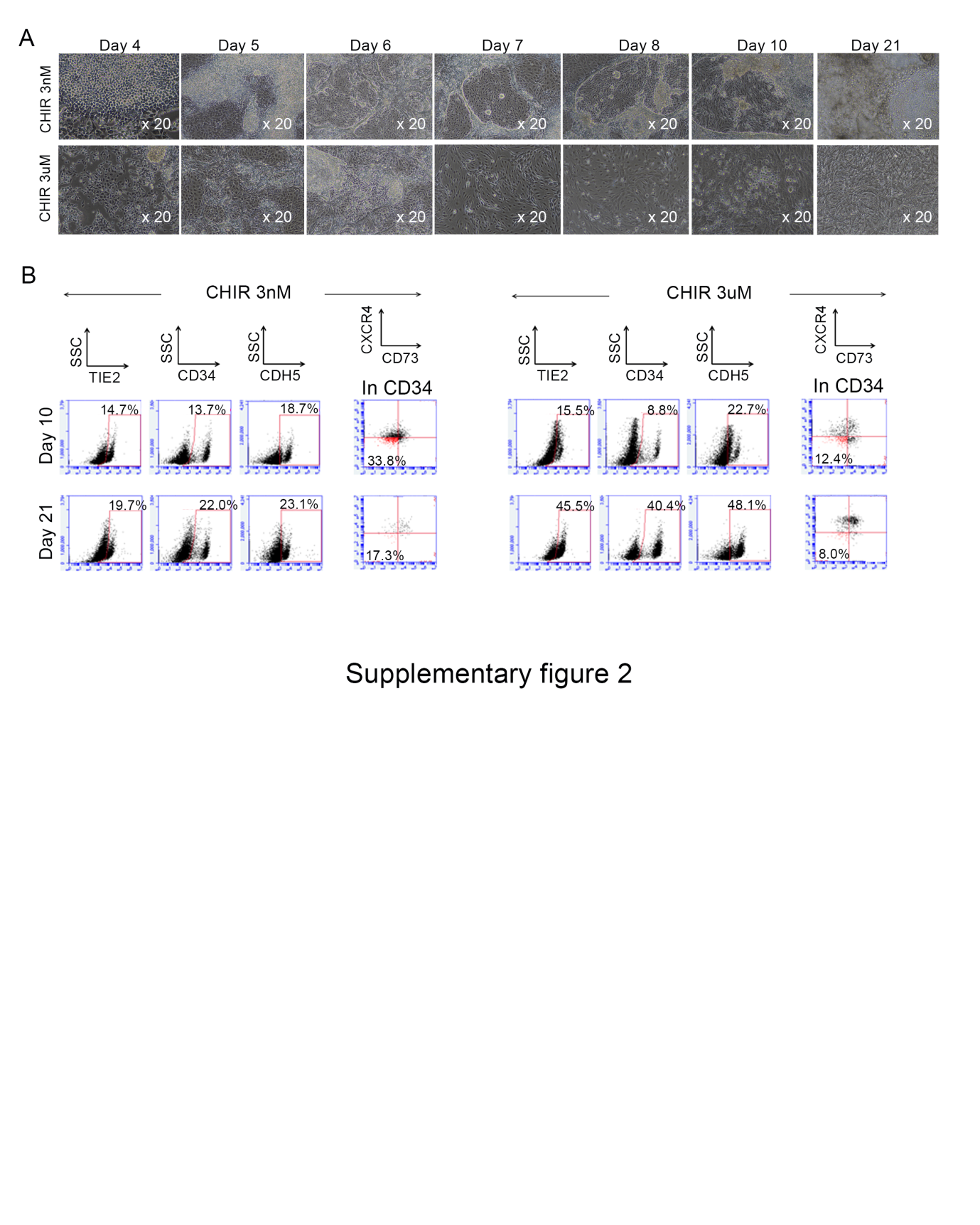


**Supplementary figure 2.** HE from CHA52 ESCs was induced by low concentration of CHIR99021 (3nM), but not high concentration (3µM) and successfully generated hematopoietic lineage cells. **(A)** The representative morphology of PSC-derived HE according to the concentration of CHIR99021. Magnification, 20×. **(B)** Proportions of TIE2, CD34, and CDH5 was examined using differentiated HE cells by CHIR. Red colored plot indicates CXCR-CD73- cells in CD34^dim^.


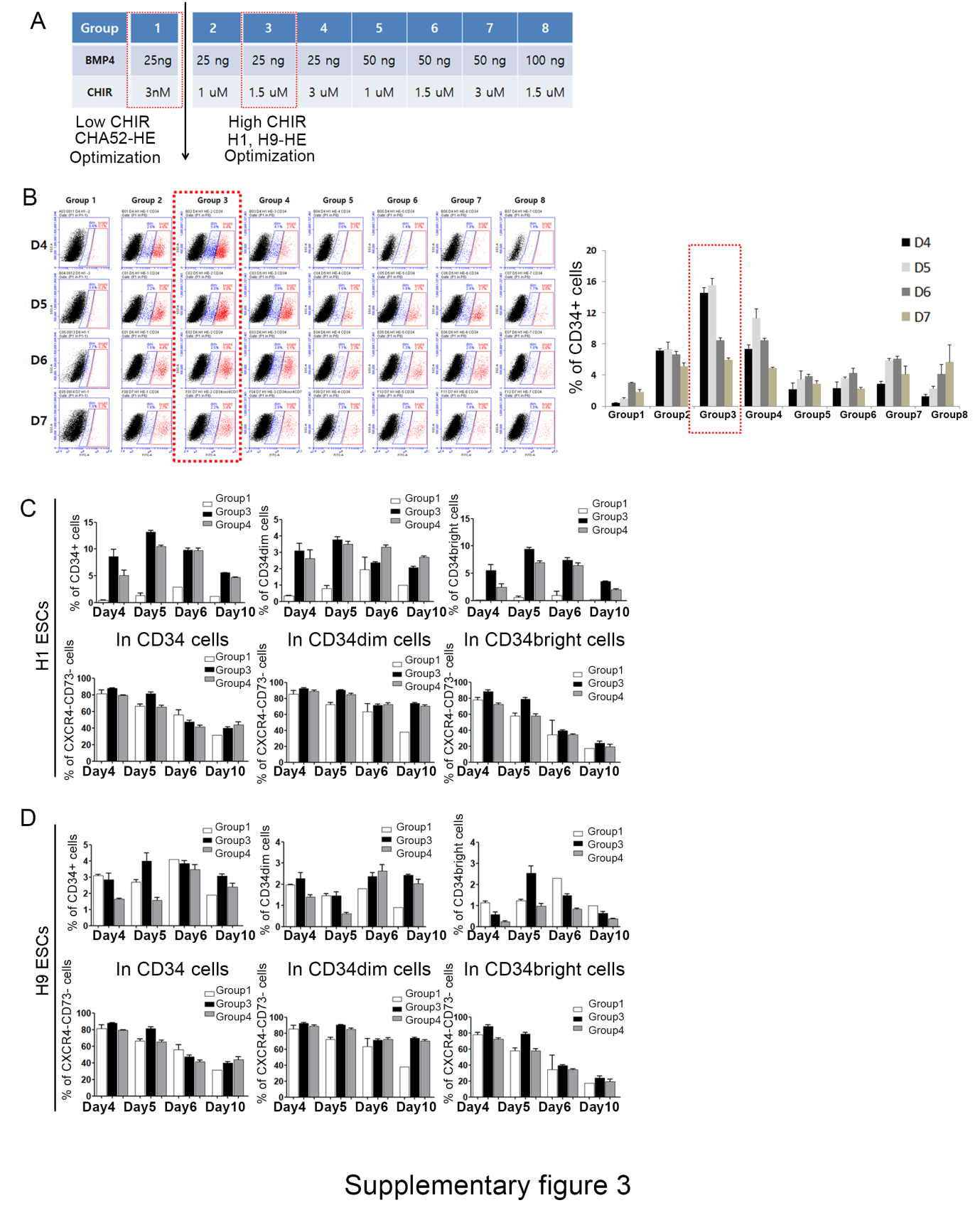


**Supplementary figure 3.** Optimization data for BMP4 (25ng, 50ng, 100ng) and CHIR99021 (3nM, 1µM, 1.5µM and 3µM) in H1 and H9 ESC-derived HE. **(A)** The 8 groups were designed to select optimization culture condition for definitive HE. Selecting low CHIR99021 (3nM) for CHA52 ESCs and high CHIR99021 (1.5μM) for H1 and H9 ESCs was optimal for inducing definitive HE via mesodermal commitment, respectively. **(B)** FACS data showed that CHIR99021 1.5μM and BMP4 25ng/ml were required to generate high CD34^+^ cells in H1 ESCs, compared to that of other groups. Data are shown as means ± SEM of at least two experiments. **(C)** Flow cytometry data showed that H1 ESC-derived CD34^+^ cells as well as CXCR4^+^CD73^+^ cells in CD34^+^ HE displayed the highest their frequency under group 3. Data are shown as means ± SEM of at least three experiments. **(D)** Flow cytometry data showed that H9 ESC-derived CD34^+^ cells as well as CXCR4^+^CD73^+^ cells in CD34^+^ HE displayed the highest their frequency under group 3. Data are shown as means ± SEM of at least three experiments.


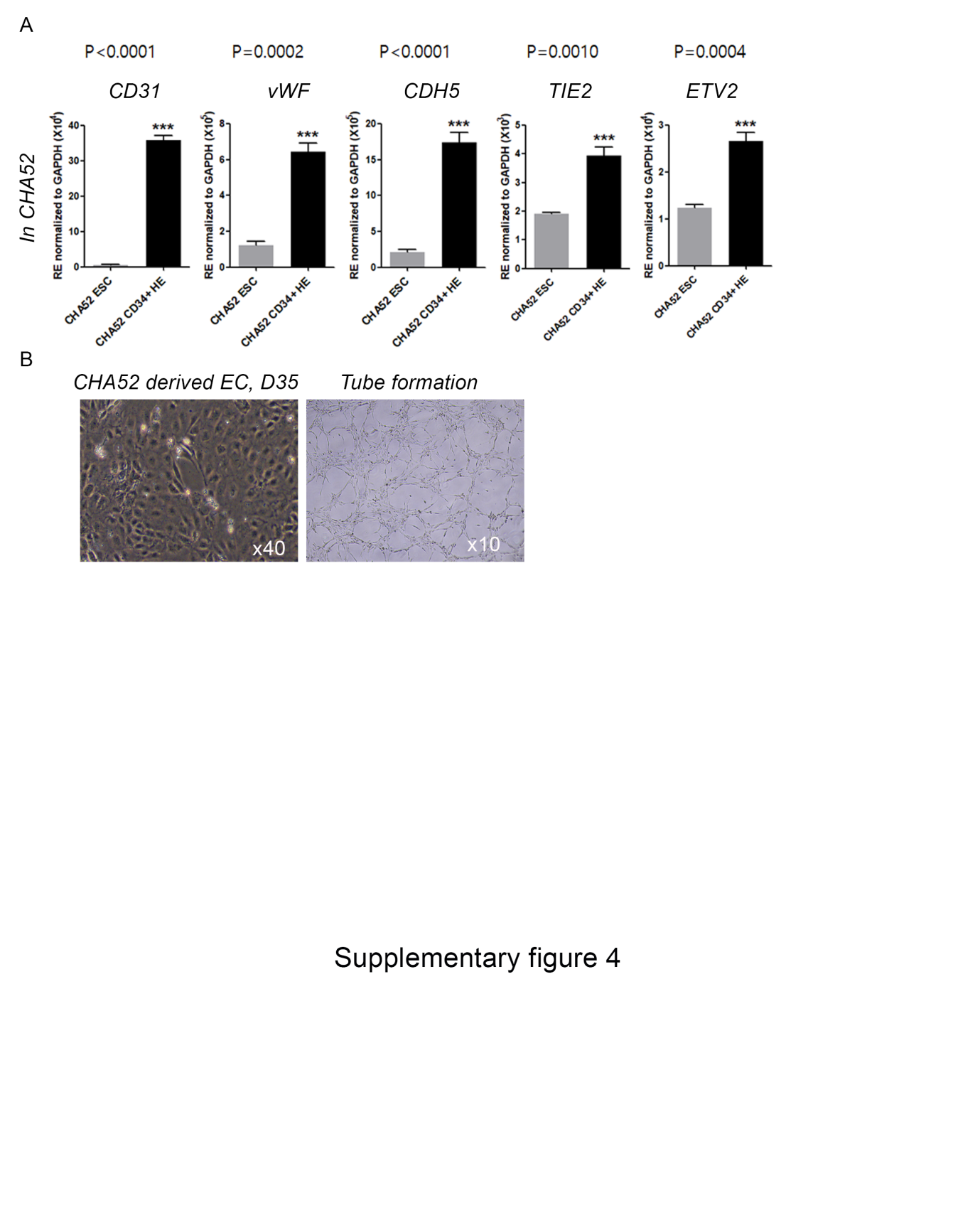


**Supplementary figure 4.** The ability of CD34^+^ HE was assessed for endothelial properties by PCR and tube formation. **(A)** After CD34 purification, qRT-PCR analysis of mRNA expression related to endothelial cells was carried out using hESC-derived CD34^+^ HEs. Data were normalized by glyceraldehyde-3-phosphate dehydrogenase (GAPDH) and expressed relatively to undifferentiated hESCs. Data are shown as means ± SEM from at least three independent experiments. (n=3)(^***^P < 0.001) **(B)** The ability of CD34^+^ HE to differentiate into endothelial cells was determined by further culturing under EGM-2 medium. These ECs formed tube immediately.


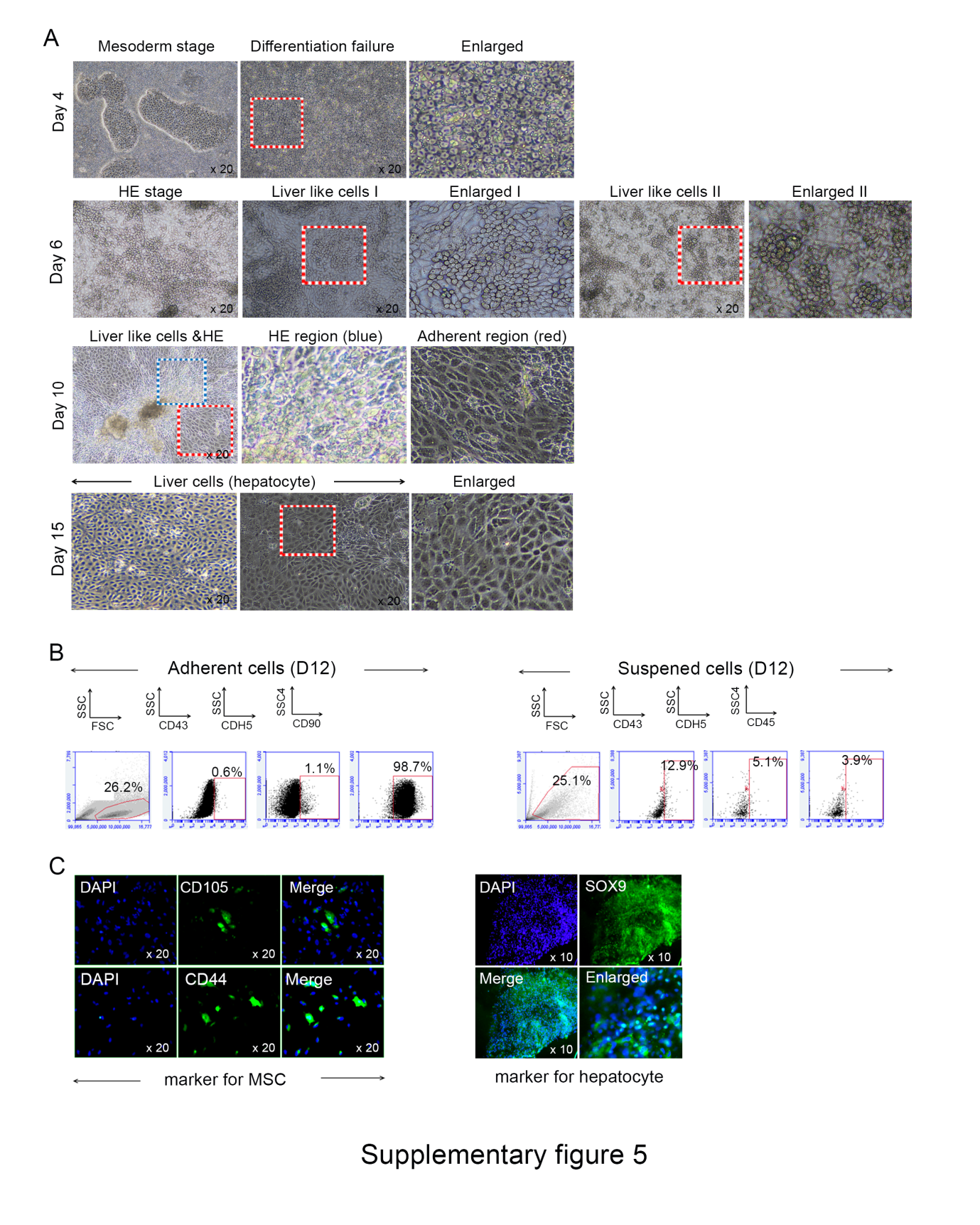


**Supplementary figure 5.** Failed HE from PSCs differentiated into hepatic like cells, which hampered HE territory maintenance. **(A)** The morphology of PSC-derived hepatic like cells was shown. These cells further differentiated into hepatocytes with formation of monolayer polygonal shaped cells. Red box includes hepatocyte. Magnification, 20×. **(B)** Proportions of CD43, CDH5, and CD45 was highly reduced, suggesting non-HE. **(C)** SOX9 was strongly expressed in adherent cells at day 15 after differentiation, implying generation of hepatocyte.


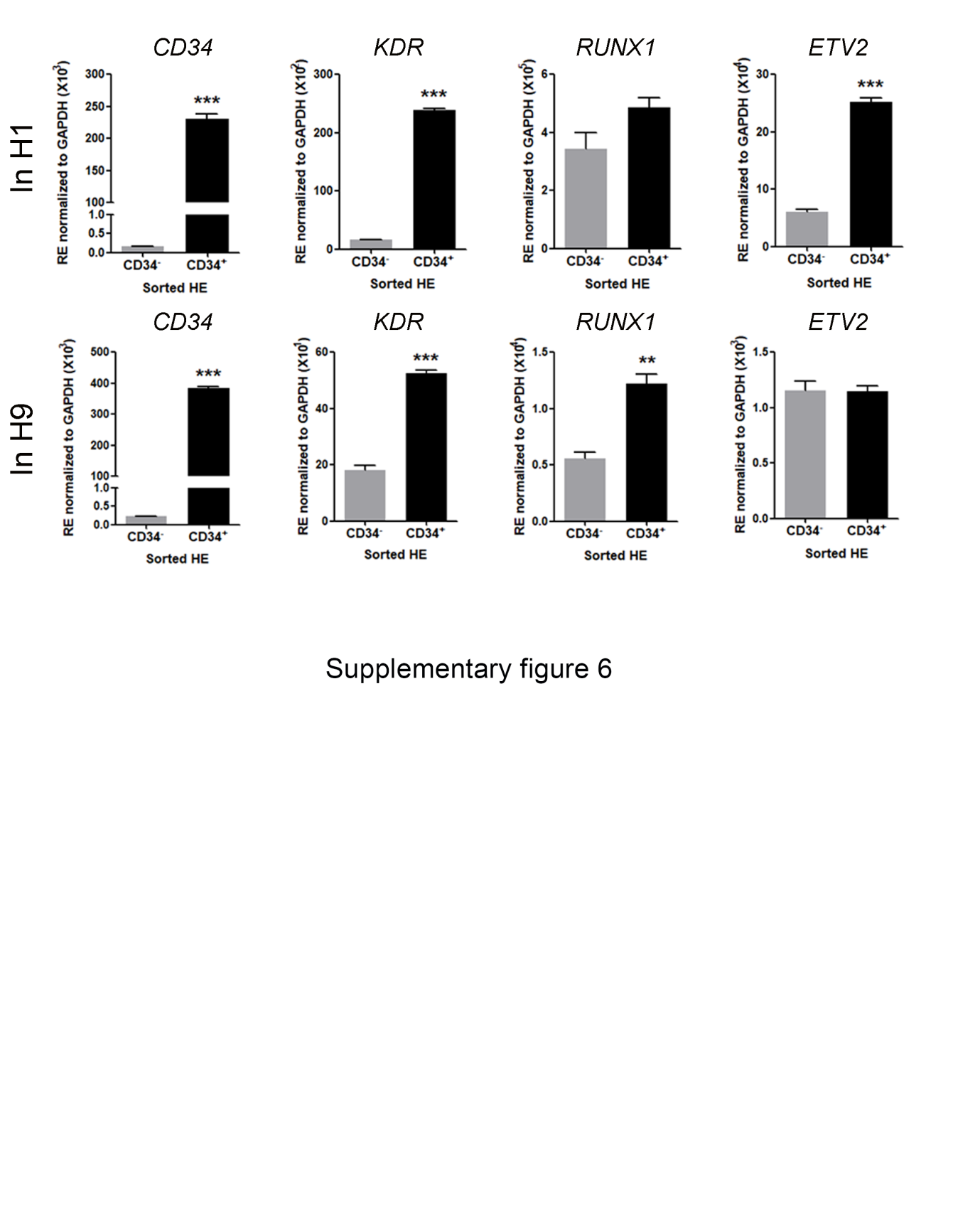


**Supplementary figure 6.** A transcript level of representative hematopoietic lineage committed factors in CD34^+^ and CD34^-^ cells was measured by qRT-PCR. Both H1 and H9 ESCs displayed the high level of genes including CD34, KDR, RUNX1, and ETV2 in CD34+ HE. Data are shown as means ± SEM from at least three independent experiments. (^**^P < 0.01, ^***^P < 0.001)


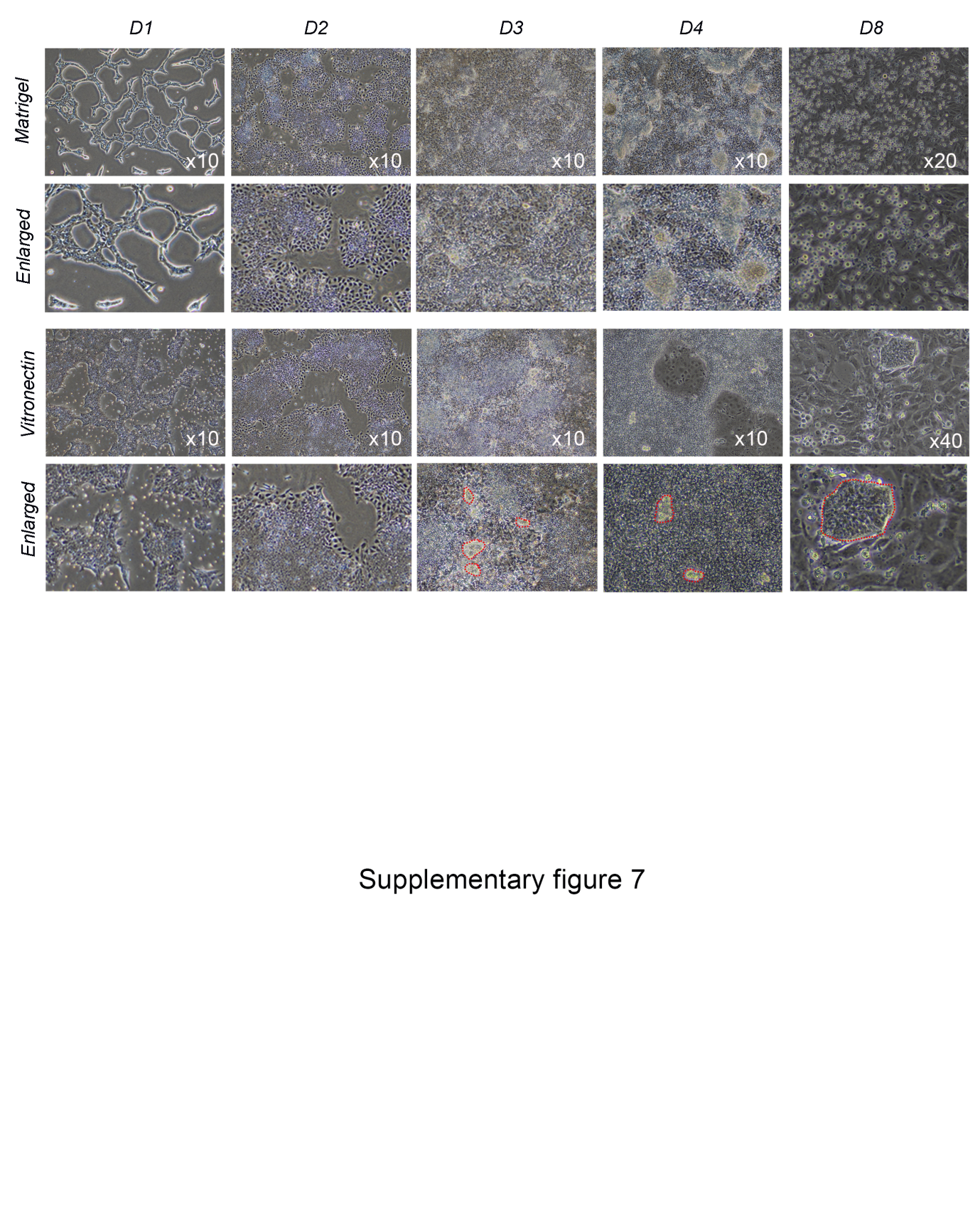


**Supplementary figure 7.** Comparison data for matrigel and vitronectin coating in differentiation into hematopoietic lineage cells from PSCs. Morphological changes indicate that matrigel is better than vitronectin to differentiate into hematopoietic lineages. Vitronectin coated culture system failed to differentiation to hematopoietic lineage cells due to hampering of remained ESCs into hematopoietic lineages. Red circle, ESC colony. Magnification 10×.
